# Recruiting a representative sample of urban South Australian Aboriginal adults for a survey on alcohol consumption

**DOI:** 10.1186/s12874-020-01067-y

**Published:** 2020-07-06

**Authors:** KS Kylie Lee, Michelle S. Fitts, James H. Conigrave, Catherine Zheng, Jimmy Perry, Scott Wilson, Dudley Ah Chee, Shane Bond, Keith Weetra, Tanya N. Chikritzhs, Tim Slade, Katherine M. Conigrave

**Affiliations:** 1 Faculty of Medicine and Health, Discipline of Addiction Medicine, NHMRC Centre of Research Excellence in Indigenous Health and Alcohol, The University of Sydney, King George V Building, 83-117 Missenden Road, Camperdown, NSW 2050 Australia; 2 Centre for Alcohol Policy Research, La Trobe University, Bundoora, Victoria 3084 Australia; 3 Menzies School of Health Research, Alice Springs, Northern Territory 0870 Australia; 4 Aboriginal Drug and Alcohol Council SA, Underdale, South Australia 5032 Australia; 5 Watto Purunna Aboriginal Primary Health Care Service, Adelaide, South Australia Australia; 6 National Drug Research Institute, Curtin University, Shenton Park, Western Australia 6102 Australia; 7 Faculty of Medicine and Health, Matilda Centre for Research in Mental health and Substance Use, The University of Sydney, Camperdown, New South Wales 2050 Australia; 8 Sydney Local Health District, Royal Prince Alfred Hospital, Drug Health Services, Camperdown, New South Wales 2050 Australia

**Keywords:** Aboriginal, Torres Strait Islander, Australia, Methodology, Alcohol, Recruitment, Representative, Population survey, Prevalence

## Abstract

**Background:**

Population estimates of alcohol consumption vary widely among samples of Aboriginal and Torres Strait Islander (Indigenous) Australians. Some of this difference may relate to non-representative sampling. In some communities, household surveys are not appropriate and phone surveys not feasible. Here we describe activities undertaken to implement a representative sampling strategy in an urban Aboriginal setting. We also assess our likely success.

**Methods:**

We used a quota-based convenience sample, stratified by age, gender and socioeconomic status to recruit Indigenous Australian adults (aged 16+) in an urban location in South Australia. Between July and October 2019, trained research staff (*n* = 7/10, Aboriginal) recruited community members to complete a tablet computer-based survey on drinking. Recruitment occurred from local services, community events and public spaces. The sampling frame and recruitment approach were documented, including contacts between research staff and services, and then analysed. To assess representativeness of the sample, demographic features were compared to the 2016 Australian Bureau of Statistics Census of Population and Housing.

**Results:**

Thirty-two services assisted with data collection. Many contacts (1217) were made by the research team to recruit organisations to the study (emails: *n* = 610; phone calls: *n* = 539; texts *n* = 33; meetings: *n* = 34, and one Facebook message). Surveys were completed by 706 individuals – equating to more than one third of the local population (37.9%). Of these, half were women (52.5%), and the average age was 37.8 years. Sample characteristics were comparable with the 2016 Census in relation to gender, age, weekly individual income, Indigenous language spoken at home and educational attainment.

**Conclusion:**

Elements key to recruitment included: 1) stratified sampling with multi-site, service-based recruitment, as well as data collection events in public spaces; 2) local services’ involvement in developing and refining the sampling strategy; and 3) expertise and local relationships of local Aboriginal research assistants, including health professionals from the local Aboriginal health and drug and alcohol services. This strategy was able to reach a range of individuals, including those usually excluded from alcohol surveys (i.e. with no fixed address). Carefully pre-planned stratified convenience sampling organised in collaboration with local Aboriginal health staff was central to the approach taken.

## Background

Population estimates of alcohol consumption are used to inform funding and design of initiatives to prevent and treat unhealthy alcohol use (drinking above recommended guidelines, including alcohol use disorders). However, for Aboriginal and Torres Strait Islander (Indigenous) Australians, methods employed and findings from these population surveys have been called into question [1, 2]. Estimates from the largest and often quoted national survey of Indigenous Australians, for example, have been shown to underestimate alcohol consumption in women by up to 700% and in men by up to 200% [2]. A recent meta-analysis has found that across different studies, estimates of drinking risk vary greatly within and between Indigenous Australian communities [3]. While considerable variation no doubt exists, uncertainty arises because of limitations to sampling methods. There is a need for accurate population estimates of alcohol consumption among Australia’s First Peoples, as the process of colonisation has exposed that community to additional risk of harms from alcohol [4].

Recruiting a representative sample of harder-to-reach or marginalised population for surveys on alcohol consumption (or other health risk behaviours) is difficult in any community [5–7]. Sampling strategies designed for mainstream populations may not be effective or appropriate in Indigenous populations. Also, for Indigenous Australians, a complex interplay of political, legislative and discriminatory factors makes alcohol a more sensitive topic to enquire about [8, 9]. For example racist stereotyping of Indigenous Australians being “drunks” [9] and fears of child removal policies [10] can increase barriers to accurately answering questions about alcohol in surveys [11].

Common recruitment strategies used by national household surveys in Australia include postal surveys, random telephone dialling or door knocking at private dwellings [1, 12]. However, Indigenous Australians may be less likely to have land lines than other Australians [13]. Also, sampling approaches which rely on residential dwellings may miss individuals residing in short stay caravan parks, hotels, hostels, ‘town camps’ or those ‘living rough’ [2, 6, 14]. Without access to residential locations (typically used by large population surveys) [15–17] extra care must be taken in site selection to ensure participants reflect the target population.

Some surveys of urban Indigenous Australians have used stratified, multi-stage sampling to identify districts where the density of households with Indigenous Australians is high [18]. From there a random selection of households within each district was contacted (in a general health survey) [18]. However, in some Indigenous communities door knocking is considered inappropriate, especially when asking about sensitive topics such as dementia [13] or alcohol. Another survey used repeated convenience sampling at community events (in urban, rural and remote settings) to ask young Indigenous Australians about sexually transmissible infections and blood-borne viruses (‘GOANNA survey’) [19]. It recruited 2.6% of the total estimated resident Aboriginal population, the largest known representative survey of Indigenous Australians (aged 16–29).

Multi-pronged, stratified sampling to recruit a representative sample has been described in surveys on health (Australia) [13, 20–23] and self-identity/values (Canada) [24, 25]. Such an approach attempts to recruit participants from a range of sites or services where the desired demographic of individuals may be located or attend. In addition, community engagement in health surveys such as: endorsements from local Indigenous Australian organisations; employing local Indigenous Australian research assistants as interviewers and translators [14, 26–29]; and afterwards, disseminating findings back to participants and local services [26], have been shown to improve participation.

In an urban Indigenous Australian context, some surveys on alcohol and other drugs [29] or on general health [13, 22] have worked with local services to develop lists of household members in that community. In a study of alcohol and other drug use in young people (aged 8–17), convenience sampling was used to recruit 95% (*n* = 105/110) of eligible individuals [29]. However, it was not clear how the list of individuals was generated. Another study of young people’s health recruited just over half (56%) of the target sample using a convenience sampling frame, developed with groups of young people themselves [22]. The ‘Koori Growing Old Well’ study (on dementia) achieved a representative sample compared with relevant census data [13]. They did this by using a three-stage sampling frame: 1) generating a list of community members from Aboriginal community controlled health service partners, which was augmented by 2) snowball sampling, and then also by 3) comparison with known census data. This last example represents one of the best approaches in our opinion of an attempt to recruit a representative sample of harder-to-reach populations in a health study.

In this paper, we present and assess a novel comprehensive sampling strategy in the context of surveying alcohol use status among urban Indigenous Australians. We aimed to determine whether, compared to official census reports, our sampling strategy produced a demographically and socioeconomically representative sample of an urban Indigenous community in the Australian state of South Australia (SA). We also summarised activities taken to ensure community support and advice, an appropriate sampling frame and site selection.

## Methods

Study methods were designed by investigators in consultation with the Aboriginal Drug and Alcohol Council of SA (ADAC). Ethical approval was obtained from the Aboriginal Health Council SA (AHCSA; Ref: 04/15/621) and (as this study was part of a larger survey of alcohol consumption) from Metro South Health Human Research Ethics Committee in Queensland (Ref: HREC/16/QPAH/293).

### Setting

The urban community was defined by an ‘Indigenous area’ used by the Australian Bureau of Statistics 2016 Census of Population and Housing [30]. It is located within the capital city of Adelaide in the state of South Australia. Its exact location is not revealed to protect the confidentiality of the community. Around 2% of the total adult population (aged 16+) who live in this urban community are Aboriginal and/or Torres Strait Islander individuals [30].

### Eligibility

Community members were invited to participate if they met inclusion criteria (i.e. being of Aboriginal and/or Torres Strait Islander descent and aged 16 yrs.+). Participants were required to reside in the nominated community (‘local council’) boundary area unless they were homeless or living in a hostel.

### Recruitment site selection

Local organisations which served Indigenous Australian clients within the council area were identified and invited to be recruitment sites for the study. Local study investigators advised that current Census methods to recruit participants would not be feasible for a survey on a sensitive topic like alcohol in this urban site (e.g. selecting every ‘n’th house, calling land line phone numbers, door knocking) [11]. Accordingly, identified organisations were asked to nominate other relevant services or public locations where recruitment could be conducted using a form of snowball sampling. In some cases, services which were unable to take part in data collection still referred other services.

### Sample stratification

No local list of eligible participants was available from a local land council or community controlled health service. So, we developed a sampling frame including age group (16–24, 25–44, 45–64, 65+ years), gender and socioeconomic status categories (full time employment, students, unemployed, homeless) of the local Indigenous population to match the most recent census data reported for the target ‘Indigenous Area’ [30].

Based on a survey of alcohol use among urban Indigenous Australians from 1994 [27] widely accepted as the most ‘reliable’ estimate available [1], we expected 50% prevalence of current alcohol misuse (i.e. exceeding National Health and Medical Research Council [NHMRC] limits) among the Indigenous population. We estimated therefore that a sample size of 581 would yield overall estimates with relative standard errors of 1%. We then increased the target sample size to 700. This was equivalent to more than one third of the local Indigenous Australian population and of ample magnitude to generate reliable estimates.

To generate strata targets, the sample size was multiplied by the proportions of males and females who were full-time workers, students, unemployed, or homeless as indicated from census data. Homelessness was defined as individuals living at no fixed address or in supported accommodation (boarding houses, hostels), ‘couch surfing’, sleeping in a car or ‘living rough’.

### Target quota for each site

Sites were allocated recruitment targets using custom software written in R. Demographic information about individuals accessing local services was obtained from each organisation to generate a table of potential participants. For example, if an organisation reported that they currently served thirty men aged 16–24 who were unemployed, then these participants would be added to the table of potential participants in the appropriate age and employment status cells. The program then randomly sampled from this table of potential participants until each row of the desired stratified sample was allocated to an eligible site. For example, university events would be eligible to be allocated the portion of the desired sample expected to be full-time students. Portions of the population which did not fall into a stratification target (e.g. part-time employment) were allocated to sites likely to contain general community members (e.g. shopping malls or public events like local festivals). Daily review of the recruitment progress against the sampling frame during data collection (by KL, MF, JC) helped ensure any instances of inadvertent over or under sampling could be corrected for.

Service providers and community groups were grouped for recruitment site allocation according to an over-arching service ‘type’. The ‘type’ of services included: Indigenous Australian health service; community group; Indigenous Australian-specific adult education; for the unemployed; for public housing recipients; homelessness; mental health; alcohol and other drug service (including diversion program); childcare; hobby group; cultural group; and public event.

### Project promotion

The research team consulted with the local study site for 9 months prior to commencement of data collection. This period was set aside to: 1) enable time for the research team to develop and strengthen relationships with local service providers and community groups; 2) enable the research team to respond accordingly to local recruitment suggestions; and 3) give local organisations enough time to organise a letter of intent to promote study recruitment to their clients or community group (an ethical requirement).

Recruitment days were advertised by each service or community group via posters and verbal promotion by service staff. The lead project officer (MF) liaised with service staff and coordinators to organise data collection times.

There were two broad types of data collection activities: 1) recruitment events that coincided with existing programs and services; and 2) other events such as paid stalls at local shopping centres or festivals, a specifically designed ‘family fun day’ hosted by a local primary health care service, ‘ad hoc’ visits to local ‘parklands’, beaches, services frequented by individuals who were ‘living rough’, shopping centres or skate parks. In some cases, staff suggested that research assistants base themselves at their service for a period of time (e.g. half a day; once off or multiple visits).

### Data collection

Data collection was conducted from July to October 2019 by a team of up to 10 research assistants (7 Aboriginal, 3 non-Indigenous; 6 male and 4 female). The Aboriginal research assistants were either health professionals working in drug and alcohol service delivery and advocacy (*n* = 1), general health practitioners (*n* = 3), a student enrolled in a research masters (n = 1) or medicine (n = 1), a research assistant (n = 1). Four team members had lived and worked in the study site for more than 25 years each. Three of the Aboriginal research assistants usually worked in one recruitment site. Potential participants in this site were reassured that they were free to decline taking part and that this would not affect their relationship with the service in any way. The non-Indigenous staff were project officers (*n* = 2) and a study investigator (n = 1). One day face-to-face training in study methods and survey administration was provided to all research assistants (June 2019; facilitated by KL and KC). Survey participants were asked to complete a survey once and received a $20 supermarket voucher as reimbursement for their time.

### Survey instrument

Data collection was performed using an interactive tablet computer-based application ‘The Grog Survey App’ [31] (herein referred to as the ‘App’) developed as part of a larger study [31, 32]. The App has been shown to be an accurate [32] and acceptable tool [33] compared to a clinical assessment conducted by an Aboriginal health professional [32]. The development of the App and composition of its survey items have been described elsewhere [31] (see supplementary material for survey items). Broadly, the App features questions on demographics, alcohol consumption (10-items), alcohol dependence (3-items based on ICD-11 [34]), harms to self or others, treatment access and participants’ feedback on using the App. The App includes culturally appropriate questioning style and gender-specific voice and images, and, ‘reads out’ the questions in English or Pitjantjatjara (an Aboriginal language spoken in a region that intersects South Australia, Western Australia and Northern Territory).

Data reported on here include time taken to complete the survey (in minutes) and participant demographics (age, gender, median weekly income, Aboriginal or Torres Strait Islander language spoken at home; and highest level of schooling completed [primary or secondary; up to year 12]). Tablet computers were synchronised daily to a secure encrypted University of Sydney server to enable data transfer and monitoring of demographics of those recruited.

### Documentation of recruitment activities

Researcher interactions by two coordinating staff (MF, KL) with local service providers and community groups were documented in two spreadsheets. The first sheet included all contacts made with local services to recruit services as potential sites and during data collection itself (i.e. emails, phone calls, face-to-face or web meetings, text messages). Data recorded for each interaction included: date, length and type of interaction, name of key contact, and any key challenges. The second sheet documented all engaged organisations, and information or how they were referred to the research team, and if they ultimately participated in data collection.

### Data analysis

All data analysis was conducted in R. To assess the representativeness of the sample, we compared demographic features of the observed sample to what was expected based on the national Census for Population and Housing (2016) [30]. Sample features assessed were age, gender, median weekly income (for each individual), if an Aboriginal or Torres Strait Islander language was spoken at home, and highest level of schooling completed. Population pyramids were constructed to compare the age and gender composition of the sample to the 2016 census. T-tests were used to determine whether organisations that participated in data collection received more contacts from the research team than organisations that did not take part in data collection up to the time of agreeing or declining participation. Agreement to participate was defined by receipt of a letter of support for the study from that organisation (an ethical requirement).

## Results

### Data collection

#### Recruitment of organisations

**Fig. 1:**
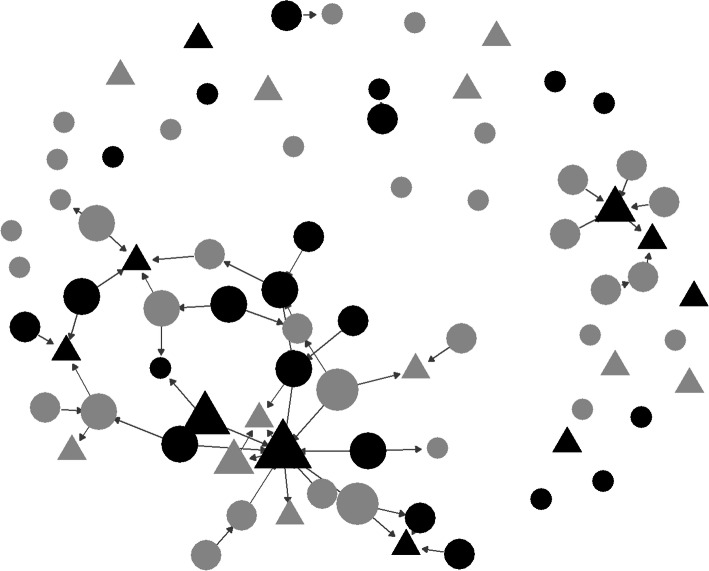
Referrals between organisations. Black nodes indicate organisations who participated in data collection. Nodes are sized relative to the number of other organisations they referred

Twenty-one local services were initially identified by the research team. These services identified other services. In total 77 services were approached to take part in the study. Thirty-two services (41.5%) ultimately took part in data collection. Half of these services (50.0%, 16/32) also referred other services. Many (42.2%, 19/45) organisations that did not ultimately participate in data collection assisted in recruitment of other services to the study (Fig. 1).

Many contacts (1217) were made in order to recruit organisations. Most contacts were emails (*n* = 610), followed by phone calls (*n* = 539), texts (*n* = 33), meetings (*n* = 34), and one Facebook message. Organisations who ultimately participated in data collection received similar numbers of contacts prior to agreeing to take part relative to those who did not (22.10 vs 17.22; t = − 1.03, df = 37.21, *p* = 0.31; across all communication types). Similarly, there was little difference in the average number of hours spent on the phone to organisations between participating and non-participating services (0.95 vs 0.75; t = − 0.98, df = 35.99, *p* = 0.33).

#### Data collection

Data collection occurred on 36 days over a period of 3.7 months (July, September, October 2019). The App survey was commenced 730 times. In 24 cases the survey was not completed. This was due to the participant leaving due to time commitments (*n* = 12), technical problems (*n* = 4), or discomfort with App content (*n* = 8). Total minutes spent on the App varied widely (min. = 4.30; max. = 78.60). The average duration was 14.77 min (*SD* = 6.33). The final sample size was 706 (37.9% of the eligible local Indigenous Australian population).

Participants were mostly recruited from public places such as shopping malls or community events (Table 1). Participants were also recruited from services for harder-to-reach groups (e.g. individuals who were homeless or attending a mental health, or alcohol and other drug service).

**Table 1:** The sample we aimed to recruit from each site

Site	Number	Percent
Public space	440	62.3
Indigenous health service	68	9.6
Community groups	51	7.2
Indigenous community colleges	43	6.1
Unemployment and housing services	34	4.8
Homelessness services^a^	34	4.8
Mental health and AOD^b^ services	25	3.5
Childcare services	7	1.0
Cultural groups	4	0.6

Public spaces included shopping malls and public events such as local festivals; ^a^ Local Aboriginal research assistants assigned clients to the homelessness strata based on their local knowledge of participants surveyed at hostels for individuals living rough, when accessing a food van, a health service or community centre, or living rough (in local ‘parklands’); ^b^ alcohol and other drug services

While most participants lived in town, a small proportion reported living in local parkland or scrub (i.e. outdoors; Table 2).

**Table 2:** Location where participants reported living

Location	Number	Percent
Town or city	629	89.1
Indigenous community	40	5.7
Parkland or scrub	10	1.4
Other	27	3.8

#### Comparison to the 2016 Australian census of population and housing

**Fig. 2:**
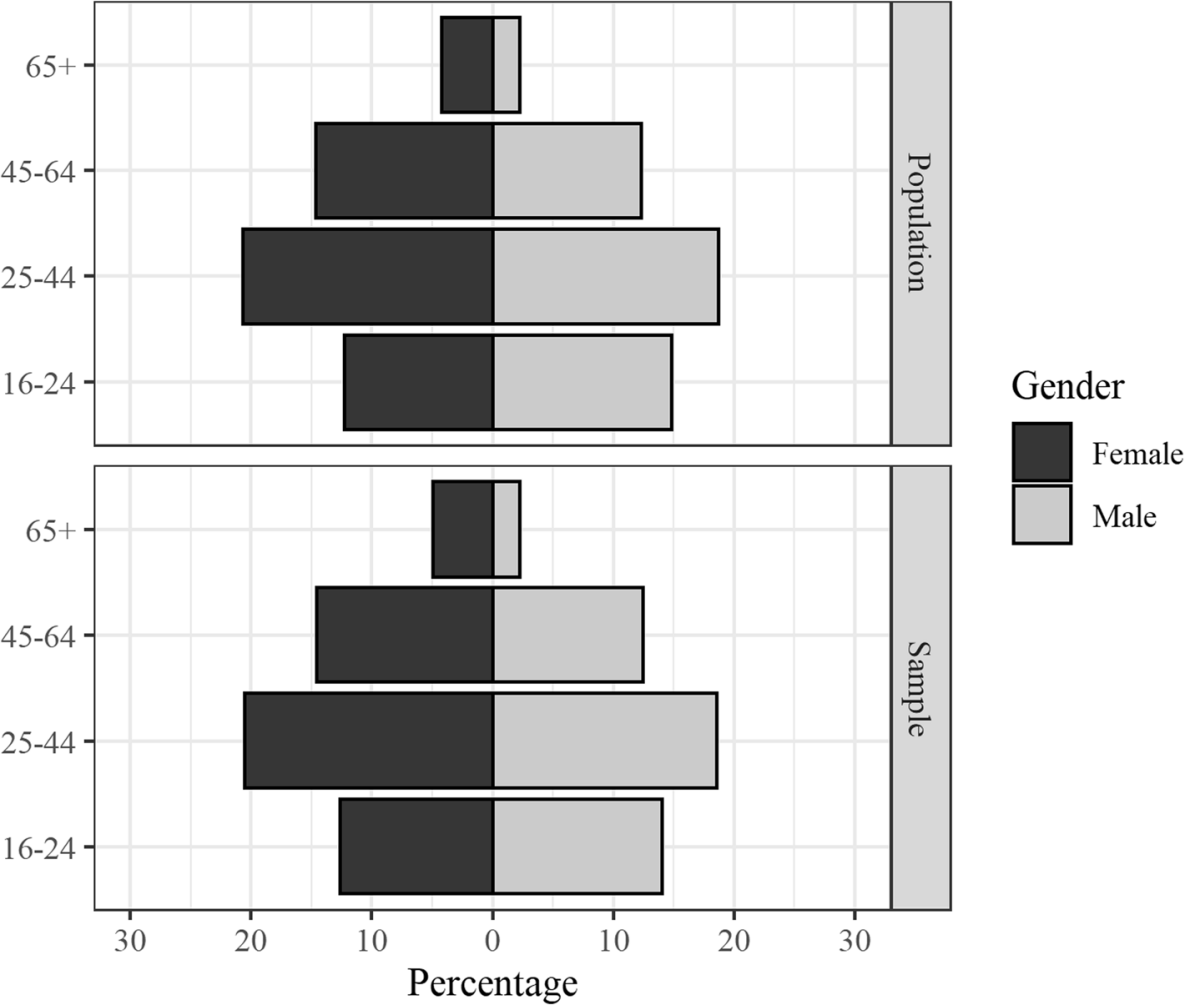
Comparison of population and sample demographic make-up. The male population is displayed on the right side of the figure. Females are displayed on the left with darker bars. Population data taken from 2016 Census of Population and Housing

**Fig. 3:**
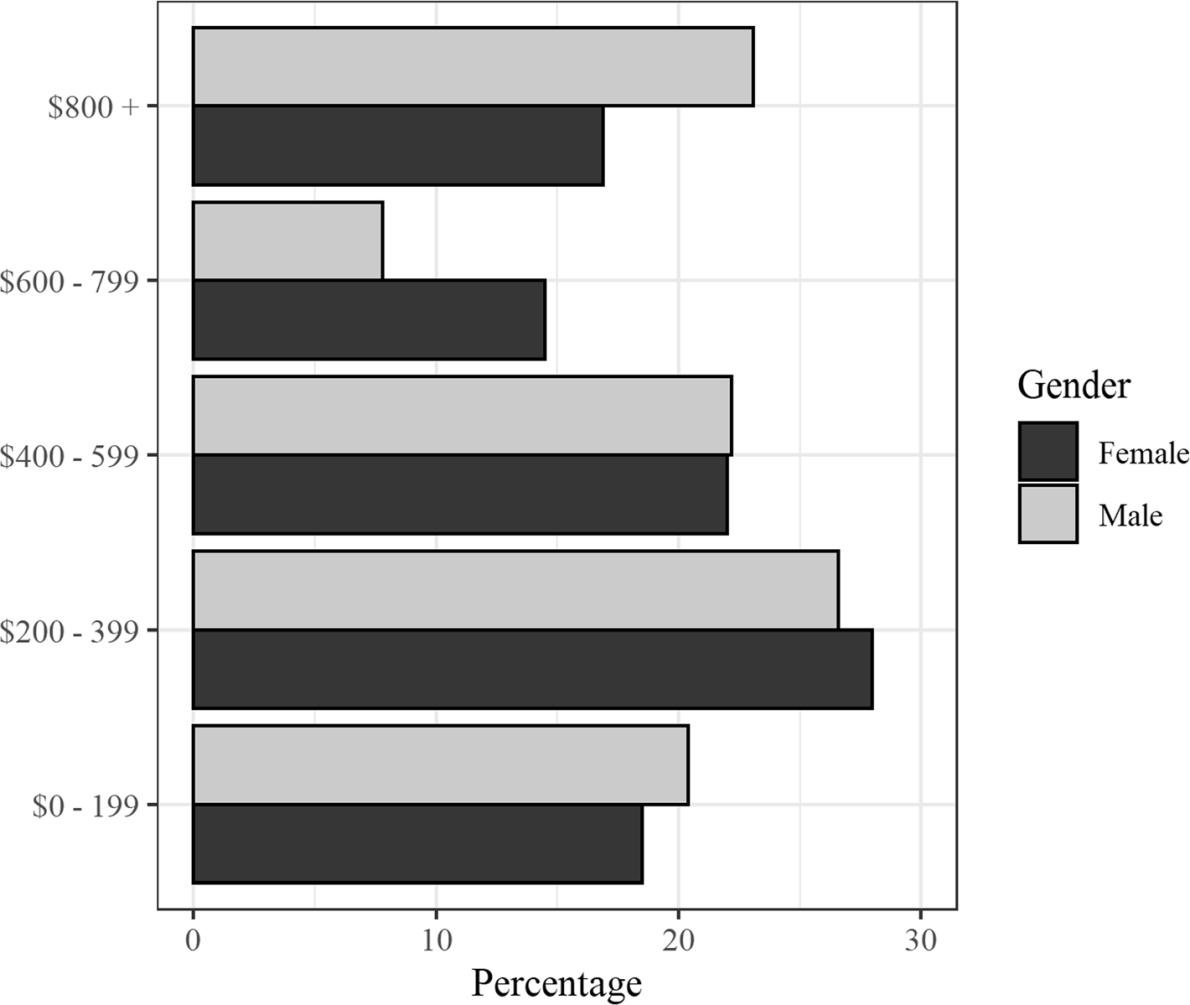
Money earned per week: percentage of respondents by gender. Females are indicated with darker bars. Males are indicated with lighter bars

As planned, the numbers of males and females in each age category matched census data closely (Table 3; Fig. 2). The average age of participants was 37.9 years. Just over half of participants were female, *n* = 383 (52.5%). The median individual income reported by both male and female participants was $400–599 (Fig. 3). This is consistent with the Census’ estimate of weekly personal income for this local population ($420) [30]. From the App, 6.37% of participants speak an Indigenous Australian language at home. This is consistent with Census data (6.70%). From the App, three quarters (75.21%) of the sample had completed Year 10 of high school. This is slightly lower than Census data (80.27%).

**Table 3:** Sample gender and age composition relative to targets

Age	*n*	sample %	census %	difference^a^ %
Female				
16–24	89	12.61	12.24	0.37
25–44	145	20.54	20.67	−0.13
45–64	103	14.59	14.65	−0.06
65+	35	4.96	4.24	0.72
Male				
16–24	99	14.02	14.87	−0.85
25–44	131	18.56	18.73	−0.18
45–64	88	12.46	12.35	0.12
65+	16	2.27	2.25	0.01

^a^ Absolute difference between percentages

## Discussion

Using a sample framework stratified by age, gender and socioeconomic status, and quotas for convenience recruitment within this framework, we were able to achieve a representative sample for this harder-to-reach population [30]. Demographic characteristics closely matched available census data for the local community. This included matching for individual weekly income and Indigenous Australian language spoken at home, variables that were not used in the stratification. The sample size recruited was equivalent to 37.9% of the target population [30]. Comprehensive stratified multi-site sampling may be a useful technique for achieving a representative sample for this or other harder-to-reach populations [21, 24].

### Sampling a range of unique spaces

We demonstrated that a representative sample could be achieved by working closely with local organisations. Individuals were then recruited from a range of sites including spaces operated by community organisations, and from public event and local festivals. We also conducted ‘ad hoc’ visits to local skateparks, shopping strips, beaches and ‘parklands’ (where people were known to be ‘living rough’) [24]. This multi-pronged approach gave the research team access to a range of participants. Using just one type of approach (i.e. service-based or via local events/spaces exclusively) could have resulted in individuals being missed [5, 19]. Bypassing the option of sampling individuals from residential addresses provided a culturally respectful approach (personal communication with S Wilson and J Perry). It also allowed the research team access to marginalised community members in this urban setting (e.g. those living in hostels, living rough or referred to a court diversion program). These individuals are typically overlooked in large population surveys [2, 6] and this may be one reason that surveys of alcohol consumption typically provide underestimates compared to sales data [35].

### Drawing on local knowledge

Working with local services to refine the sampling frame gave the research team access to unique local knowledge of the target population. It also likely helped the study participants feel safe, with data collection conducted in the same environment where they had established connections with service staff [36]. Services provided up-to-date information about current client populations and their likely whereabouts [18]. Local information was used to inform the design of recruitment events (i.e. via bespoke morning teas or ‘family fun days’ [22], barbeques, or by linking in with existing programs). This was further enhanced by having four Aboriginal staff on the team of research assistants who were well known in the study community. To further increase access to data collection events, transport to the recruitment site was provided for some harder-to-reach participants (e.g. younger and older individuals) [6].

### Reaching harder-to-reach populations

Successful recruitment elements of similar multi-pronged sampling efforts include surveying from multiple services or public spaces [23–25], ‘ground-up’ involvement from local services before and during data collection [13, 20–25], and employment of local Aboriginal research assistants [13, 20, 22–24]. Despite these common elements, previous studies have achieved varied success. One survey [21] did not assess the representativeness of their sample, while the sample characteristics of other studies [20, 23–25] did not match available census or other national data. Some studies did not report their target sample size [20, 24, 25], while others did not reach their target sample size [21, 22].

Our sampling method relied on an available estimate of population characteristics. This was supplemented by consultation with local study investigators (SW, JP) and health service representatives (DA, SB, KW). In our situations, such an estimate is not available. This requires other sampling methods such as respondent-driven and time-location sampling. These were deemed not suited to the needs of this population or context for several reasons. Time-location sampling would require a weighted sample [37], which we felt would not give a true snapshot of the urban site (i.e. it would not use all the data collected). This method would also make it harder to describe the findings back to the study community. Instead, elements of time-location sampling were incorporated. For example, research assistants frequented areas during “busy” hours to conduct data collection (e.g. pay day at a local shopping centre).

Respondent-driven sampling was deemed not suitable for a range of reasons. Firstly, we had a small team of field research assistants. Data collection did not occur every day or in a regular location. So, it was not possible to nominate a guaranteed and regular location where our research assistants would be for individuals to complete the survey. Secondly, local study investigators advised that this approach could be burdensome on some participants (i.e. individuals would need to hold onto a token, then pass it to other individuals, who would then locate the team to complete a survey). They advised that it would be better if we went to places where participants were likely to be. Thirdly, recruiting via respondents can cause under-or over-representation of certain population sub-groups [38]. Hence, previous research has recommended a tailored approach, particularly in diverse communities [39, 40].

### Implications for policy, practice and research

This sampling approach could be valuable not just for other Indigenous Australians but in other contexts, such as developing countries. If there is no census data available to guide recruitment, an alternative estimate of sample composition may be required, for example, estimates based on observation [41]. Alternatively, for other harder-to-reach populations, such as people who inject drugs, a range of methods, such as ‘capture recapture’ are used to assess population characteristics [42]. To implement the approach taken, sufficient resources are needed to tailor recruitment events to the local context and ensure appropriate training and mentoring for research staff. A diverse team of engaging and highly skilled research assistants who were known to local services and community members appeared to increase the study’s accessibility to the target population. The electronic survey platform, with regular syncing to a secure server, also offered flexibility in survey administration. It also allowed for daily monitoring of recruitment progress against targets or for recording reasons for attrition in real time. It is possible that the anonymous and confidential survey administration approach may also have increased acceptability of survey participation on this potentially sensitive topic.

### Limitations

Because of the need to preserve anonymity, participants’ names were not recorded. It is possible that some participants took the survey more than once. To reduce the likelihood of this, core researchers attended every event and greeted participants. We strived to access as many public spaces as possible. However, some types of services were not included because of practical constraints (e.g. hospitals, youth detention and correctional services; where approval processes proved challenging). Effort was made to recruit individuals with similar characteristics to those groups (e.g. from local court diversion programs, hostels, shopping centres, clinics and public events). Despite efforts to recruit a broader sample, it was interesting that our sample had similar demographics to the Australian Bureau of Statistics 2016 Census. However, there is a lot of variation within the category of ‘unemployed’ in terms of lifestyles including drinking patterns. Based on consultation, we are confident that we have captured the real-life diversity, including harder-to-reach sectors of the community, and in approximately the proportions that they are estimated to be present. We based the sampling frame and site targets on census data as it is the most comprehensive comparison data available in an Australian context. However, we know that the census itself may fail to recruit harder-to-reach individuals. Our sampling frame and recruitment approach was similar to the Koori Growing Well Old study [13]. However, our sampling frame was solely drawn by referencing census population data (as we were unable to generate a list of individuals’ names). Our sampling strategy was heavily reliant on community-based organisations and public places, so individuals who were not accessing these services or who avoided public places may not have been reached. We tried to avoid over sampling of individuals using services as these participants may have higher perceived health or social needs. It was for that reason that we also recruited from settings like local parklands, skateparks or shopping centres. Data collected on the number of contacts made with local services to organise recruitment was only recorded by the two key coordinating staff (MF, KL). It did not include recruitment efforts by other research assistants or local study investigators and so is an underestimate of the true effort involved. We did not systematically collect reasons why some services did not ultimately participate in data collection or why some individuals chose to not take part in the study. The recruitment processes described are based on a single urban Indigenous Australian community. While elements of the approach could be useful to other urban Indigenous Australian communities, the community at this study site had longstanding professional and personal relationships with the research assistants and local study investigators which is likely to have enhanced recruitment success.

## Conclusion

The sampling framework and recruitment approach used in this study was able to achieve a sample comparable with the most recent Australian Bureau of Statistics census data for this same geographical area. The sampling strategy was able to reach populations often excluded from national household surveys on alcohol (i.e. people with no fixed address). Three elements were key to the recruitment approach taken: 1) a stratified sampling strategy comprised of multi-site service-based recruitment alongside data collection events held in public spaces (planned and ‘ad hoc’); 2) involvement of local services to develop and refine the sampling strategy; and 3) expertise of highly skilled local Aboriginal research assistants, including health professionals from local Aboriginal health and drug and alcohol services. This approach is likely to have relevance to recruiting other harder-to-reach populations around the world, including on surveys on sensitive topics.

## Supplementary information

**Additional file 1.** Grog Survey App survey items.

## Data Availability

Data for this project is stored at the University of Sydney based at Drug Health Service, KGV Building, Missenden Road, Camperdown New South Wales, 2050 Australia. Contact A/Prof Kylie Lee for more information (kylie.lee@sydney.edu.au).

## Abbreviations

ADAC: Aboriginal Drug and Alcohol Council South Australia
AHCSA: Aboriginal Health Council of South Australia
DA: Dudley Ah Chee
HREC: Human Research Ethics Committee
Indigenous Australian: Aboriginal and/or Torres Strait Islander peoples
JC: James H Conigrave
JP: Jimmy Perry
KC: Katherine M Conigrave
KW: Keith Weetra
KL: KS Kylie Lee
MF: Michelle S Fitts
SW: Scott Wilson
SB: Shane Bond
SA: South Australia
