# Supplementary material for: Recruiting a representative sample of urban South Australian Aboriginal adults for a survey on alcohol consumption
Source: BMC Med Res Methodol. 2020 Jul 6;20:183. doi: 10.1186/s12874-020-01067-y (PMC7339418; doi:10.1186/s12874-020-01067-y)
Supplement: Supplementary file 1 — Additional file 1. Grog Survey App survey items. [file 12874_2020_1067_MOESM1_ESM.pdf]

Grog Survey App

24/06/2020

Introduction

RA

1. Choose community location

<Free text>

2. Please select the participant's gender

<Male>

<Female>

3. Is the participant

<Aboriginal>

<Torres Strait Islander>

<Aboriginal and Torres Strait Islander>

<non-Indigenous>

4. How did the participant hear about us?

<Facebook>

<Poster/postcard>

<Friend or family>

<Researcher>

<Local service>

<Saw us>

<Never heard of us before>

<Other>

5. Where is the participant being surveyed?

<Clinic or other treatment centre>

<Shop or post office>

<At a house>

<Park, bush, beach>

<School or community college>

<Festival or community event>

<Youth or community centre>

<Detention centre or prison>

<Other>

6. The survey is ready for the next person to complete. Pass it to them now.

<NA>

Consent

7. Please select the language for audio

<English>

<Aboriginal (Pitjantjatjara)>

8. You are invited to help us try out this iPad App. It makes it easier for people to talk about their drinking.

You can stop at any time and don't have to tell us why.

Your answers are private, which means when we use your answers, we won't write down who you are. We may also use your answers for future research.

<NA>

9. Do you want to take part in this study?

<Yes>

<No>

10. You don't want to complete the app survey. Please give this iPad back to the person who handed it to you.

<NA>

Demog

11. If you need help, ask the person who handed you this iPad. First we'll ask some questions about you.

<NA>

12. How old are you?

<{Slider 16 to 100}>

13. When is your birthday?

<{select [month/day]}>

14. Where do you usually live?

<Town or city>

<Aboriginal community>

<Outstation>

<Parkland or scrub>

<Other>

15. What is your post code?

<{4 digits number (postcode)}>

<I don't know>

16. What is your suburb or community?

<Free text>

17. What language do you speak at home?

<English>

<An Aboriginal or Torres Strait Islander language>

<Another language>

18. How far did you go in school? (Just tell us the highest year you finished)

<I've never been to school>

<Year 1>

<Year 2>

<Year 3>

<Year 4>

<Year 5>

<Year 6>

<Year 7>

<Year 8>

<Year 9>

<Year 10>

<Year 11>

<Year 12>

19. Have you done any training or study after school (choose any that is right for you)

<Training>

<University>

<No, I didn't do any training or study after school>

20. Training

<I'm doing TAFE or apprenticeship now>

<I started but did not finish TAFE or apprenticeship>

<I completed TAFE or apprenticeship>

<Training through CDP>

21. University

<I am studying at Uni now>

<I started but didn't finish a degree at uni>

<I finished a degree at uni>

22. Do you have a paid job?

<Yes>

<No>

23. Can you tell me more about your paid job?

<I work full-time>

<I work part-time>

<I work some of the time (casual)>

<I'm on Work for the Dole or CDP>

<Other>

24. Do you have any other ways of getting money? (You can choose more than one)

<No – I look after the kids>

<No – I don't get any money>

<Royalites, corporations or gate money>

<I'm on Centrelink – like the dole, family allowance or a pension>

<From family>

<From friends>

<Other>

25. How much money do you earn each week? (this is the amount you receive as cash or into your bank account)

<0-$199>

<$200-$399>

<$400-$599>

<$600-$799>

<$800 or more>

<I don't want to answer>

26. How much money do you earn each fortnight? (this is the amount you receive as cash or into your bank account)

<$0-$399>

<$400-799>

<$800-1199>

<$1200-1599>

<$1600 or more>

<I don't want to answer>

27. On a day when you drink, how much money do you spend on grog?

<$0 - $25>

<$26 - $50>

<$51 - $75>

<$76 - $99>

<$100 or more>

<I don't want to answer>

28. Is there any chance you could be pregnant now?

<Yes>

<No>

29. Do you use condoms, the rod, the pill or something from the doctor to stop you getting pregnant?

<Yes>

<No>

Alcohol

Consumption

30. Thanks. Now I'm going to ask you about any grog you might drink.

<NA>

31. Some people drink grog most days, while others drink 'once in a blue moon'. How often have you had any grog in the last 12 months?

<Never>

<Once in a blue moon (less than once a month)>

<Sometimes (2-4 times a month)>

<2-3 times per week>

<Most days or every day>

32. Can I check, in the last 12-months (since [event name] last year), at special events like sporting carnivals, weddings or funerals, have you had any grog at all?

<Yes I've had some grog in the last 12 months>

<No, I had no grog at all in the last 12 months>

33. In your life, have you ever had any grog at all?

<Yes>

<No>

34. Can you tell me one type of grog that you drink most often?

<Beer>

<Full-strength beer>

<Mid-strength beer>

<Low-strength beer>

<Wine>

<Red wine>

<White wine>

<Sparkling wine>

<Spirits>

<Straight spirits>

<Mixed drinks>

<Port or sherry>

<Cider>

35. How much grog do you have on a typical day when you drink? Choose whichever answer is closest.

<1 or 2 standard drinks>

<3 or 4 standard drinks>

<5 or 6 standard drinks>

<7, 8 or 9 standard drinks>

<10 or more standard drinks>

36. How often would you drink 'this much grog or more' in one day (24 hours)?

<Never>

<Once in a blue moon (Hardly ever, less than once a month)>

<Sometimes (1-3 times a month)>

<Weekly>

<Most days or every day>

37. Can I ask you about the last day you had any grog. Where were you drinking? (you can choose more than one)

<House, camp>

<Scrub, bush>

<Park, beach>

<Hotel, club, pub or bar (licensed premises)>

<Restaurant/café>

<Car or other vehicle>

<Other>

38. When was the last day you had any grog? (Choose the last day when you had a drink)

<grog calendar>

39. How many people were you drinking grog with? (Dag how many people were in your drinking circle)

<{Slider to choose how many people in drinking group - from "just you" to "9+ other people"}>

40. What types of grog were you drinking that day? (Choose all types of grog you drank that time)

<Beer>

<Wine>

<Port or sherry>

<Spirits>

<Cider>

<Other>

41. What types of [grog type] were you drinking that day?

<{grog drinks guide}>

42. What did you mostly drink [grog type] out of?

<{grog container guide}>

43. How much grog did you put in it?

<{slider to fill container}>

44. How much [grog type] did you drink that last day?

<{slider to select grog quantity}>

45. Just want to check, is this what you had or what the whole group had?

<Just me>

<The group>

46. How many people were you drinking grog with? (Dag how many people were in your drinking circle)

<{Slider to choose how many people in drinking group - from "just you" to "9+ other people"}>

47. On that day was that ALL YOU had to drink?

<Yes, that's all I had to drink>

<No, I drank other grog too>

48. What else were you drinking that day?

<Beer>

<Wine>

<Port or sherry>

<Spirits>

<Cider>

<Other>

49. Thanks. Before that, when was the last day you had any grog. Can you show me when that was?

<grog calendar>

50. Thanks. Before that, when was the last day you had a drink?

<grog calendar>

51. What about the time before that?

<grog calendar>

52. Thanks. People drink different amounts of grog at different times. Pick any of these that are true for you. (you can choose more than one)

<I drink more when I'm happy (sporting carnivals, weddings, parties)>

<I drink more when times are bad (stressing out, funerals, feeling alone)>

<I drink more when I'm bored>

<I drink more when there's more money>

<My drinking never changes>

53. Some people have times when they don't drink at all. In the last 12 months, [since RELEVANT EVENT last year], have you had a time when you had no grog at all?

<Yes>

<No>

54. How long was your longest time of not drinking in the last 12 months?

<1 day>

<2 days>

<3 days>

<4 days>

<5 days>

<6 days>

<1 week>

<2 weeks>

<3 weeks>

<4 weeks>

<5 weeks>

<6 weeks>

<7 weeks>

<2 months>

<3 months>

<4 months>

<5 months>

<6 months>

<7 months>

<8 months>

<9 months>

<10 months>

<11 months>

55. Which of these reasons explains why you didn't drink? (you can choose more than one)

<Health reasons>

<Because of family and friends>

<Because of court or prison>

<Grog costs too much>

<Because of where I was living>

<Other reasons>

56. Health reasons (you can choose more than one)

<I got really sick>

<I want to be healthy>

<I've been hurt by someone else's drinking>

<The doctor or health staff told me to stop or slow down>

<I was in rehab or detox>

<I got pregnant>

<Someone close to me got pregnant>

<Other>

57. Because of family and friends (you can choose more than one)

<Death or illness in the family>

<I saw arguments or fights when people drink>

<Caring for kids or grandkids>

<To be a good example for the kids>

<Someone close to me got pregnant>

<Other>

58. Because of court or prison (you can choose more than one)

<I had court coming up>

<I stopped when I was in prison>

<I stopped while I was on parole>

<Other>

59. Because of where I was living (you can choose more than one)

<I moved to a different community>

<Alcohol restrictions in my area>

<I started spending time with different people>

<Other>

60. Which of these reasons explain why you never had a drink?

<Health reasons>

<Because of family and friends>

<I have seen bad examples of what grog can do>

<Grog costs too much>

<It's hard to get grog where I live>

<Other reasons>

61. Health reasons (you can choose more than one)

<I want to be healthy>

<I was worried I might get problems with grog or be an 'alcoholic'>

<I was pregnant or might get pregnant>

<I've been hurt by someone else's drinking>

<My health is bad for other reasons, and I can't drink>

<Other reasons>

62. Because of family and friends (you can choose more than one)

<I wanted to be there to help family>

<I saw arguments or fights when family drink>

<To be a good example for the kids>

<I got pregnant or wanted to get pregnant>

<People around me don't drink>

<Other>

63. Other reasons (you can choose more than one)

<I'm not interested in drinking>

<I've no particular reason for not drinking>

<Because of my culture>

<Because of my religion>

<I don't like the taste>

<Other>

64. People stop drinking grog for different reasons. Which of these reasons explain why you stopped drinking? (you can choose more than one)

<Health reasons>

<Because of family and friends>

<Because of court or prison>

<Grog costs too much>

<Because of where I was living>

<Other reasons>

65. Health reasons (you can choose more than one)

<I got really sick>

<I want to be healthy>

<I've been hurt by someone else's drinking>

<The doctor or health staff told me to stop or slow down>

<I was in rehab or detox>

<I got pregnant>

<Someone close to me got pregnant>

<Other>

66. Because of family and friends (you can choose more than one)

<Death or illness in the family>

<I saw arguments or fights when people drink>

<Caring for kids or grandkids>

<To be a good example for the kids>

<Someone close to me got pregnant>

<Other>

67. Because of court or prison (you can choose more than one)

<I had court coming up>

<I stopped when I was in prison>

<I stopped while I was on parole>

<Other>

68. Because of where I was living (you can choose more than one)

<I moved to a different community>

<Alcohol restrictions in my area>

<I started spending time with different people>

<Other>

Dependence

69. Thanks for telling me about how much you drink. Some people drink grog because they want to. Some people drink because they need to. These next questions are about what it's like for you in the last 12 months [since EVENT NAME last year]

<NA>

70. Some people feel like grog is the boss of them. How often do you feel grog makes all the decisions? (so you could not stop drinking, even if you tried)

<Never>

<Once in a blue moon (Hardly ever, less than once a month)>

<Sometimes (1-3 times a month)>

<Weekly>

<Most days or every day>

71. Some people's hands shake when they stop drinking or before their first drink of the day. In the last 12 months, how often does this happen to you?

<Never>

<Once in a blue moon (Hardly ever, less than once a month)>

<Sometimes (1-3 times a month)>

<Weekly>

<Most days or every day>

72. Some people spend more time drinking than doing other things they need to do, like looking after family, culture or work. In the last 12 months, how often does this happen to you?

<Never>

<Once in a blue moon (Hardly ever, less than once a month)>

<Sometimes (1-3 times a month)>

<Weekly>

<Most days or every day>

Harms

73. You have not had any grog in the past 12 months. But maybe people around you drink. In the last 12 months [that is, since... insert relevant known community event/holiday... last year] has any drinker given you worries? (You can choose more than one)

<No>

<Yes, a drinker has hit me or got in a fight with me>

<Yes, my money runs out because it goes on someone's grog>

<Yes, the kids in my house have been scared by a drinker>

<Yes, other worries like stress>

74. In the last 12 months, how often has a drinker hit you or been in a fight with you?

<Once in a blue moon (less than once a month)>

<Sometimes (1-3 times a month)>

<Weekly>

<Most days or every day>

75. In the last 12 months, how often have you been to hospital or clinic because you got hit or in a fight with a drinker?

<Once in a blue moon (less than once a month)>

<Sometimes (1-3 times a month)>

<Weekly>

<Most days or every day>

76. In the last 12 months, how often did your money run out because it all went on someone's grog?

<Once in a blue moon (less than once a month)>

<Sometimes (1-3 times a month)>

<Weekly>

<Most days or every day>

77. In the last 12 months, how often have kids in your house been scared by a drinker?

<Once in a blue moon (less than once a month)>

<Sometimes (1-3 times a month)>

<Weekly>

<Most days or every day>

78. In the last 12 months, how often have you felt stressed out by a drinker?

<Once in a blue moon (less than once a month)>

<Sometimes (1-3 times a month)>

<Weekly>

<Most days or every day>

79. The next few questions ask about when you used to drink.

<NA>

80. Back when you drank grog, did your drinking hurt you or hurt someone else? (You can choose more than one)

<No>

<Yes, I got in a fight>

<Yes, I fell down>

<Yes, I hit someone or something>

<Yes, all my money went on grog>

<Yes, the kids in my house were scared when I was drinking>

<Yes, I got into trouble with police or security guards>

81. Back when you drank grog, you told us you got in a fight. Did you need to go to the hospital or clinic?

<Yes>

<No>

82. How many times did you go to the hospital or clinic because you got into a fight when you were drunk?

<1 time>

<2 times>

<3 or more times>

83. Back when you drank grog, you told us you fell down. Did you need to go to hospital or clinic?

<Yes>

<No>

84. How many times did you go to hospital or clinic because you fell down when drinking?

<1 time>

<2 times>

<3 or more times>

85. Back when you drank grog, how often would money run out because it went on grog?

<Once in a blue moon (less than once a month)>

<Sometimes (1-3 times a month)>

<Weekly>

<Most days or every day>

86. Back when you drank grog, how often did kids in your house get scared by your drinking?

<Once in a blue moon (less than once a month)>

<Sometimes (1-3 times a month)>

<Weekly>

<Most days or every day>

87. Back when you drank grog, how often did you get into trouble with police or security guards?

<Once in a blue moon (less than once a month)>

<Sometimes (1-3 times a month)>

<Weekly>

<Most days or every day>

88. Grog sometimes gives people worries or problems. In the last 12 months [that is from after [event name] last year until now] has grog given you any of these problems? (You can choose more than one)

<Someone hit me>

<I fell down>

<I had a road accident>

<My money runs out because it goes on grog>

<The kids in my house get scared by my drinking>

<I get into trouble with police or security guards>

<No>

89. In the last 12 months, did you end up in hospital or clinic because you got hit when drinking?

<Yes>

<No>

90. How many times did you go to hospital or clinic because you got hit when drinking?

<1 time>

<2 times>

<3 or more times>

91. In the last 12 months, did you end up in hospital or the clinic because you fell down when drinking?

<Yes>

<No>

92. How many times did you go to hospital or clinic because you fell down when drinking?

<1 time>

<2 times>

<3 or more times>

93. In the last 12 months, how often do you run out of money because it all goes on grog?

<Once in a blue moon (less than once a month)>

<Sometimes (1-3 times a month)>

<Weekly>

<Most days or every day>

94. In the last 12 months, how often do kids in your house get scared by your drinking?

<Once in a blue moon (less than once a month)>

<Sometimes (1-3 times a month)>

<Weekly>

<Most days or every day>

95. In the last month 12 months, how often do you get into trouble with police or security guards when drinking?

<Once in a blue moon (less than once a month)>

<Sometimes (1-3 times a month)>

<Weekly>

<Most days or every day>

96. Sometimes, when people drink, they end up using other drugs like ice, heroin, pills or gunja. How often has this happened to you in the last 12 months?

<Never>

<Once in a blue moon (Hardly ever, less than once a month)>

<Sometimes (1-3 times a month)>

<Weekly>

<Most days or every day>

Treatment

97. Thanks for all your answers so far. The next questions are about getting help for drinking

<NA>

98. Have you ever got help about drinking – like from a clinic, counsellor, rehab, detox or hospital?

<Yes>

<No>

99. What made you get help?

<It was my choice>

<Family or friend pushed me>

<Someone official pushed me (like welfare, police, or courts)>

100. If someone around here wants to give away their drinking or cut down, how easy is it to get help ?

<Very easy, there is lots of help available>

<Easy, there is some help available>

<Not sure what help is available>

<Hard, there is not much help available>

<Very hard, there is no help available, or it's a long way from here>

101. How far does a person need to travel to get to a rehab to stop drinking

<Slider from 10m to 3000km>

<I don't know>

102. Can a person get a medicine to stop the grog shakes from a doctor around here?

<Yes>

<No>

<I don't know>

103. If a person is finished with the grog shakes and wants to stay away from grog, can a doctor around here give them medicine to help?

<Yes>

<No>

<I don't know>

104. If a friend or family wants to cut down or stop drinking, where should they get help? (you can choose more than one)

<Aboriginal alcohol and drug worker>

<Aboriginal health or mental health worker>

<Nurse or doctor>

<Counsellor>

<Friend, family>

<Elder>

<Minister or Pastor>

105. Where else could they get help? (you can choose more than one)

<Day centre>

<Detox or rehab>

<Hospital>

<AA or SMART Recovery groups>

<Men's or Women's groups>

<Internet or phone helpline>

<Other>

Feedback

Satisfaction

106. How easy was it to use this iPad to answer our questions?

<Image of big frown>

<Image of small frown>

<Image of straight face>

<Image of small smile>

<Image of big smile>

107. Was it okay for us to ask these questions?

<Image of big frown>

<Image of small frown>

<Image of straight face>

<Image of small smile>

<Image of big smile>

108. Were the questions easy to understand?

<Image of big frown>

<Image of small frown>

<Image of straight face>

<Image of small smile>

<Image of big smile>

109. Any other comments?

<free text>
